# Piloting a New Approach to the Treatment of Obesity Using Dexamphetamine

**DOI:** 10.3389/fendo.2015.00014

**Published:** 2015-02-09

**Authors:** Alison S. Poulton, Emily J. Hibbert, Bernard L. Champion, Traci L. Cook, David Alais, David S. Coulshed

**Affiliations:** ^1^Sydney Medical School Nepean, University of Sydney, Penrith, NSW, Australia; ^2^Department of Dietetics, Nepean Hospital, Penrith, NSW, Australia; ^3^School of Psychology, University of Sydney, Sydney, NSW, Australia

**Keywords:** obesity, dexamphetamine, weight loss, dose titration, appetite

## Abstract

**Background and aims:** There is a clear need for a new approach to the treatment of obesity, which is inexpensive and is effective for establishing lifestyle change. We conducted a pilot study to evaluate whether dexamphetamine can be used safely, combined with diet and exercise, for treating obesity. Our ultimate aim is to develop a 6-month treatment program for establishing the lifestyle changes necessary for weight control, utilizing dexamphetamine for its psychotropic effect on motivation. We viewed the anorexigenic effect as an additional advantage for promoting initial weight loss.

**Methods:** Obese adults were treated with dexamphetamine for 6 months (maximum of 30 mg twice daily), diet, and exercise. Weight, electrocardiogram, echocardiogram, and blood pressure were monitored.

**Results:** Twelve out of 14 completed 6 months treatment. Weight loss by intention to treat was 10.6 kg (95% CI 5.8–15.5, *p* < 0.001). The mean weight gain in the 6 months after ceasing dexamphetamine was 4.5 kg (95% CI 1.9–7.2, *p* = 0.003), leaving a mean weight loss at 12 months from baseline of 7.0 kg (95% CI −13.4 to −0.6, *p* = 0.03). All reported favorable increases in energy and alertness. Dose-limiting symptoms were mood changes (2) and insomnia (2). None had drug craving on ceasing dexamphetamine, and there were no cardiac complications. Among the seven women, there was a significant correlation for those who lost most weight on treatment to have the least regain in the following 6 months (*r* = 0.88, *p* = 0.009).

**Conclusion:** Our treatment with dexamphetamine, diet, and exercise was well tolerated and effective for initial weight loss. Future research will focus on identifying baseline predictive variables associated with long-term weight control.

## Introduction

Obesity is a major risk factor for diabetes, cardiovascular and liver disease, sleep apnea, some cancers, and osteoarthritis. Despite obesity research being a priority area, current treatment is having little impact at a population level as weight loss is difficult to achieve and even harder to sustain (1). Approaches to treatment such as diet, exercise, pharmacotherapy, and bariatric surgery all have their drawbacks. Bariatric surgery is becoming accepted as the treatment of choice for higher levels of obesity, but it is expensive and in some, weight may subsequently be regained (2). Pharmacotherapy can assist with weight loss and its efficacy can be enhanced by lifestyle modification (3). Although there is evidence that exercise assists with ongoing weight control (4), if medication is ceased weight is often rapidly regained at a rate proportional to the previous rate of loss (5). Continuing medication as maintenance treatment increases expense and risk of side effects. However, a proportion of individuals are able to achieve and maintain substantial weight loss (6). The way forward is to find an approach that is able to increase this proportion. Just as anorexigenic medication assists with initial weight loss, we suggest that psychotropic medication may assist with establishing the lifestyle and exercise patterns necessary for maintaining the weight loss.

Coming from a background of experience in the treatment of children with attention-deficit hyperactivity disorder (ADHD), we have observed that dexamphetamine is well tolerated and has few complications apart from consistently inducing weight loss (7). ADHD and obesity often coexist, with the characteristic impulsiveness and poor motivation in ADHD contributing to difficulty in weight control in some (8). Dexamphetamine has the therapeutic effects of enhancing cognitive control of behavior, improving motivation, and reducing impulsiveness. It is used in ADHD in combination with behavioral intervention (multimodal management) to promote behavioral change (9–11). By analogy with ADHD, we postulated that the psychotropic effects of dexamphetamine could be utilized in obesity to assist with establishing new habits to promote long-term weight control. We viewed the appetite suppressant effect as an added benefit for assisting with initial weight loss (12, 13). Dexamphetamine is associated with more weight loss than methylphenidate (14), perhaps because in addition to the dopaminergic effects it also inhibits serotonin reuptake (15). Dexamphetamine is inexpensive. We intended to use it only for 6 months, which we postulated would be long enough to establish behavioral change in a proportion of obese individuals. Despite its abuse potential, we chose to use dexamphetamine rather than a selective serotonin reuptake inhibitor (SSRI) because of its therapeutic effect on behavior, which is intrinsic to the treatment concept.

The main concerns with dexamphetamine relate to toxicity, abuse potential, and concern that the small increase in blood pressure could exacerbate ischemic heart disease (16). However, amphetamines have been previously studied for the treatment of obesity (16–19) [including in individuals with cardiovascular disease (16)] and also for their psychotropic properties (20). Griffith et al. showed that psychosis can be induced in individuals with no known predisposition within 5 days through dexamphetamine doses of 5–15 mg hourly (20). Psychosis is always preceded by an identifiable pre-psychotic phase associated with depression, followed by withdrawal and suspicion, and then loss of insight and extreme paranoia. Psychosis resolves within 1–8 h after drug withdrawal and is followed by hypersomnolence, irritability, and depression, with complete resolution after 2–3 days and none of the drug craving characteristic of detoxification from amphetamine abuse.

In the 1950s in the context of widespread prescription and misuse of dexamphetamine, there were case reports of amphetamine psychosis and malnutrition, together with depression on drug withdrawal (21). Subsequently, dexamphetamine prescribing was restricted; its main use over the past 60 years has been for treating children with ADHD. However, with the rising cost of obesity (22) and the expense of approved medical and surgical interventions (23), it is timely to question the traditional teaching that dexamphetamine is too dangerous to use for treating obesity.

There have been few recent studies that have used amphetamines for treating obesity. In 1947, Ray treated obese patients with racemic amphetamine and methamphetamine and after 8 months achieved weight losses of 12.7 kg in men and 11.0 kg in women (19). More recently, Levy et al. used stimulant medication (mainly amphetamine) in a predominantly female cohort of obese individuals, principally to treat newly diagnosed ADHD (12). They reported weight loss of 15.1 ± 10.4 kg (12.4 ± 7.2% of initial body weight) after an average of 466 days on treatment.

In 2011, Desouza et al. treated veterans with obesity and apathy with low-dose methylphenidate, to test whether it would improve motivation and assist with the lifestyle changes required for weight loss (24). Subjects were randomized to a motivation program with or without methylphenidate, or to standard nutrition counseling. Apathy scores improved comparably in all groups, with no enhancement in those on methylphenidate. However, only those on methylphenidate achieved statistically significant weight loss (average 4.6 kg over 6 months). This modest weight loss could have been due to appetite suppression. This study reported no psychotropic effects, perhaps because the dose was too low.

In the treatment of ADHD, it is important for the dose of stimulant medication to be individually adjusted to optimize behavioral functioning. The aims of this pilot study were to investigate the safety and efficacy of a 6-month period of treatment with dexamphetamine for achieving weight loss and to evaluate dose titration limited by adverse amphetamine symptoms and cardiovascular effects. The long-term aim of this research is to evaluate whether behavioral changes can be established safely using dexamphetamine and lead to maintenance of weight loss after treatment is withdrawn.

## Materials and Methods

This was an open label trial of 6 months of treatment with dexamphetamine in adults aged over 18 years with body mass index (BMI) of more than 30 kg/m^2^. Exclusion factors included the following: history of addiction to illicit drugs, uncontrolled hypertension > 140/90, symptomatic ischemic heart disease, significant kidney or liver disease, uncontrolled epilepsy, history of bariatric surgery, current treatment with psychotropic medication, systemic glucocorticoids or medication for weight loss, or family history of sudden death from cardiac causes.

At baseline, all participants had an echocardiogram and ECG to screen for cardiomyopathy, valve disease, and arrhythmia. The ECG was repeated at maintenance dose and the echocardiogram at the end of the treatment phase. Participants were monitored weekly during dose titration and monthly while on treatment. Amphetamine effects were monitored using the Amphetamine Interview Rating Scale (25).

Our study participants did not come with a diagnosis of ADHD, so there were no behavioral end points in terms of improvement in the symptoms of ADHD for guiding dose titration. We, therefore, progressively increased the dose until either the individual developed adverse effects necessitating dose reduction or the maximum formulary dose was reached. The initial dose of dexamphetamine was 5 mg at breakfast and lunchtime, followed by weekly incremental dose increases of 5 mg twice daily, to a maximum of 30 mg twice daily. Adverse effects were managed with dose reduction. At the end of 6 months, the drug was withdrawn and the participants were encouraged to maintain the exercise and dietary changes established on dexamphetamine.

The participants were asked to do 30 min of moderate exercise for at least 4 days per week and to follow a diet of 7050–7350 kJ per day, with dietician (Traci L. Cook) assessment at baseline and review after 2 months on treatment. Behavioral counseling consisted of emphasizing to the participants that the study gave them a single, unique opportunity to be treated with dexamphetamine and to use the 6 months of treatment to establish new habits for a healthy lifestyle. They were given feedback on their progress, with positive reinforcement for weight loss and reporting a favorable exercise routine.

Estimated sample size for 90% power to detect a weight loss of 4% of body weight at two-sided significance of *p* < 0.05 was 12 based on our data from children (7). The study was approved by the Nepean and Blue Mountains Local Health District Human Research Ethics Committee, and all participants gave informed consent. Medication was prospectively approved for each participant by the NSW Ministry of Health.

Statistical analysis of the changes in weight, abdominal circumference, and BMI used paired sample two-tailed Student’s *t*-tests.

## Results

Between July and November 2012, we recruited 14 participants to the study, including 2 to cover attrition (Table 1). One discontinued after 4½ months due to side effects but continued to attend reviews; the other left the study within 2 months for unrelated reasons. Her weight at her final review has been carried forward to the end of the treatment period for analysis.

**Table 1:** **Baseline data and changes in weight, body mass index (BMI), and abdominal circumference after 6 months treatment with dexamphetamine and changes in the first 6 months after ceasing dexamphetamine (between 6 and 12 months from baseline)**.

Baseline data	Women (*n* = 8)	Men (*n* = 6)	Total (*n* = 14)
	Mean ± SD	Mean ± SD	Mean ± SD
Age (years)	51.2 ± 27.2	50.2 ± 4.0	50.7 ± 20.1
Height (cm)	161.0 ± 5.0	180.8 ± 2.9	169.5 ± 10.9
Weight (kg)	95.3 ± 15.8	111.8 ± 9.9	102.4 ± 15.6
BMI (kg/m^2^)	36.7 ± 5.1	34.2 ± 3.4	35.6 ± 4.5
Abdominal circumference (cm)	106.3 ± 8.7	117.0 ± 8.3	110.9 ± 9.9

**Changes with treatment**	**Mean (95% CI)**	**Mean (95% CI)**	**Mean (95% CI)**

Weight (kg)
Baseline to 6 months	−11.9 (−20.1 to −3.7) ***p* = 0.01**	−9.0 (−15.6 to −2.3) ***p* = 0.02**	−10.6 (−15.5 to −5.8) ***p* < 0.001**
6–12 months	5.2 (0.1 to 10.4) ***p* = 0.05**	3.7 (0.9 to 6.6) ***p* = 0.02**	4.5 (1.9 to 7.2) ***p* = 0.003**
BMI (kg/m^2^)
Baseline to 6 months	−4.6 (−7.7 to −1.4) ***p* = 0.01**	−2.7 (−4.8 to −0.7) ***p* = 0.02**	−3.8 (−5.6 to −2.0) ***p* = 0.001**
6–12 months	2.0 (0.1 to 4.0) ***p* = 0.05**	1.1 (0.3 to 2.0) ***p* = 0.02**	1.6 (0.6 to 2.6) ***p* = 0.004**
Abdominal circumference (cm)
Baseline to 6 months	−9.8 (−16.5 to −3.2) ***p* = 0.01**	−8.0 (−15.3 to −0.6) ***p* = 0.04**	−8.3 (−13.1 to −4.8) ***p* = 0.001**
6–12 months	4.4 (1.0 to 7.8) ***p* = 0.02**	4.8 (−0.5 to 10.2) *p* = 0.07	4.6 (2.1 to 7.1) ***p* = 0.002**

*p = significance using two-tailed paired *t*-tests, statistically significant results in bold*.

The mean weight loss on treatment was 10.6 ± 8.4 kg, (*p* < 0.001), with seven participants (50%) losing more than 10% of their bodyweight. Weight regain in the first 6 months after stopping treatment reduced the mean weight loss to 7.0 ± 10.6 kg (*p* = 0.035), although 3 participants still recorded a weight reduction of more than 10% and a further 4 of 5–10%. One woman (who lost 31 kg on treatment) and one man continued to lose weight after ceasing dexamphetamine (6.3 and 0.4 kg, respectively, in 6 months). Of the 5 who lost more than 15% of their body weight, 3 recorded no weight regain at 3 months. Among the women, there was a significant correlation between the weight change on treatment and the weight change in the first 6 months after ceasing treatment (*r* = 0.88, *p* = 0.009), with those who lost most weight showing the least regain.

Weight loss enabled one participant with obstructive sleep apnea to cease treatment with continuous positive airways pressure and another with diabetes to have a reduction in insulin. One participant with borderline hypertension at baseline commenced antihypertensive treatment, which was continued after completing the study; another required a reduction in antihypertensives. There were no cardiac complications.

Nine participants tolerated the full dose of 60 mg/day taken as two divided doses; 4 remained on lower daily doses of 20 –50 mg. The participants’ experience of the amphetamine effects is given in detail in Table 2. This provides data that may be used to evaluate whether the widespread concern regarding the psychotropic effects of dexamphetamine justifies the perception that it is too dangerous for use in obesity. In two participants, the dose was limited due to mood changes: one was more aggressive than normal and the other more emotional and tearful. These improved with dose reduction in one and the other reduced and then ceased medication after 4½ months. Two participants took only one dose/day because of insomnia. Nine experienced a dry mouth, which was associated with ulcers in three cases. Three reported mild, transient euphoria, mainly during titration. None reported any drug withdrawal or problems with ceasing medication at the end of 6 months other than tiredness and hunger. Amphetamine effects that were perceived as beneficial were increased energy and ability to concentrate.

**Table 2:** **Number of participants with any scores (positive or negative) on the Amphetamine Interview Rating Scale at baseline, during titration, and on maintenance dose of dexamphetamine**.

	Baseline (*n* = 14)	Titration (*n* = 14)	Maintenance (*n* = 13)	Cessation (*n* = 13)
**Activation** (includes feeling active, especially alert, excited, talkative, having racing, or sexual thoughts)				
Total number experiencing activation	6^a^	14	9	6^a^
**Depressive affect**
Feel depressed	1 (−1)	5 (−0.9)	3 (− 0.3)	2 (0)
Have difficulty concentrating	2 (0)	8 (−0.4)	5 (− 0.6)	2 (0)
Have difficulty remembering	2 (0)	4 (+0.3)	3 (+ 0.3)	1 (+ 1)
Feel guilty	0	4 (−0.6)	2 (− 1)	0
Feel sad and pessimistic	3 (−0.3)	5 (−0.5)	4 (− 0.5)	1 (+ 1)
Feel tearful	1 (−1)	6 (+0.1)	4 (− 0.5)	1 (+ 1)
Total number experiencing depressive affect	3	9	6	3
**Physical symptoms**
Have blurred vision	2 (−1)	1 (+2)	3 (− 0.3)	1 (− 1)
Feel cold	1 (−1)	4 (+1)	2 (+ 1)	3 (+ 1)
Feel dizzy	2 (−2)	3 (+1)	1 (+ 1)	0
Have dry mouth	3 (−0.7)	9 (+1.4)	8 (+ 1.2)	1 (− 1)
Have a headache	3 (0)	6 (+0.6)	1 (+ 1)	3 (+ 1)
Feel hungry	0	11 (−1)	9 (− 1)	4 (+ 0.5)
Feel nauseated	2 (0)	3 (0)	2 (0)	0
Am perspiring	0	3 (+1.3)	4 (+ 1)	0
Am thirsty	1 (+1)	9 (+1.2)	8 (+ 1.2)	1 (+ 1)
Total number experiencing physical symptoms	4	13	11	5
**Euphoria** (covered by questions of feeling close to people, confident, euphoric, good overall)				
Total number experiencing euphoria	3^a^	9	7	1^a^
**Dysphoria**
Feel anxious	2 (−1)	2 (−0.5)	3 (0)	0
Feel angry	1 (−1)	3 (−1)	2 (0)	1 (+ 1)
Feel irritable	1 (−1)	5 (−0.6)	4 (+ 0.5)	1 (+ 1)
Feel less in touch with people	1 (−1)	4 (−0.4)	3 (+ 0.3)	0
Feel restless	2 (−1)	2 (0)	4 (+ 1)	1 (+ 1)
Feel strange	2 (−1)	1 (+1)	0	0
Have unusual thoughts	2 (−1)	2 (−1.5)	0	0
Total number experiencing dysphoria	2	6	8	1
**Sleepiness** (feeling sleepy or drowsy)
Total number experiencing sleepiness	2	10^a^	6^a^	6

^a^Symptoms experienced as “less than normal.”

They were asked to rate each symptom in relation to “normal” using a five-point scale from −2 to +2 (“much less than normal” to “much more than normal”) with “normal” as 0. The numbers in brackets give the mean of the symptom score for those who rated the symptom as different from “normal.”

## Discussion

In our small cohort, we have demonstrated that a relatively short period of treatment with dexamphetamine and basic behavioral intervention was effective for weight loss and was not associated with any significant complications.

The main innovation of this research is the concept of a relatively short period of treatment with dexamphetamine, utilizing the psychotropic effects on behavior. The strength was a close monitoring, which found no evidence for any concern for long-term adverse cardiovascular effects or problems with abuse or withdrawal from the drug. However, this study was unable to evaluate the long-term outcomes of multimodal management combining dexamphetamine with behavioral intervention. This was because participant numbers were restricted to the minimum required sample size due to practical considerations relating to need to establish the methodology and ethical issues regarding safety. Furthermore, behavioral management was limited, and finally, there was no control group for comparison.

Our results using dexamphetamine compare well with both previous studies using amphetamines (12, 19) and studies using other anorexigenic medications. Placebo subtracted weight losses after 24 or more weeks have been obtained as follows: dexfenfluramine 5.1 kg; phentermine with fenfluramine 9.6 kg; phentamine with topiramate 8.8 kg; sibutramine 4.2 kg; tesofensine 9.1 kg; rimonabant 4.7 kg (26). The mean weight loss on placebo in these studies was 2.4 kg. Extrapolating from this, we might reasonably anticipate a placebo-subtracted weight loss after 6 months on dexamphetamine of around 8.2 kg, which compares well and as a single drug therapy appears more effective than a SSRI. Although a placebo group was not used in this study, the lifestyle advice regarding diet and exercise in this study was basic and brief, similar to the studies referenced above, where placebo group weight loss was small. Greaves et al. conducted a systemic review of reviews of interventions on the effectiveness of dietary and physical activity interventions to prevent type 2 diabetes in adults at high risk. Greaves’ group found that weight loss of 3–5 kg was reported after lifestyle interventions over 12 months’ duration and 2–3 kg after 36 months’ duration (27).

Combining dexamphetamine with a more structured and intensive behavioral management program focusing on establishing regular exercise and eating patterns could potentially increase its efficacy for initial weight loss and for long-term weight control. We found among the women that there was a significant tendency for greater weight loss to be associated with less regain. This is contrary to the expected outcome of a simple, transient period of amphetamine-induced appetite suppression. It is, therefore, possible that our rudimentary behavioral intervention worked synergistically with the dexamphetamine for promoting greater and more sustained weight loss.

In the search for obesity treatment, the amphetamines have been largely overlooked. The argument that these are too dangerous is implausible as they have continued to be prescribed for ADHD. Increasing the therapeutic indications for dexamphetamine would certainly lead to more availability and, therefore, more potential for abuse. However, we believe that there are a large number of individuals who are struggling to lose weight and would use dexamphetamine responsibly. Careful participant selection, close observation, and relatively short treatment period would minimize the opportunity to abuse the drug and would also reduce the risks of long-term cardiovascular complications.

Our findings need confirmation with a larger, double-blind placebo-controlled study. Including a more structured behavioral management program could increase the proportion of individuals who achieve sustained weight loss. Testing for cognitive changes associated with dexamphetamine could provide a useful objective guide for dose titration. Longer studies are required for correlating baseline behavioral characteristics with long-term maintenance of weight loss, in order to be able to predict which individuals are likely to benefit most. The cost of treatment in terms of the price of medication is low. We anticipate that a well-monitored 6-month course of dexamphetamine might become first-line drug treatment for younger people at earlier stages of obesity, where concerns of cardiovascular side effects are lower due to the lower prevalence of underlying cardiovascular disease, particularly hypertension. If the results reported here can be replicated among larger cohorts, the potential impact of this treatment at a population level is enormous.

## Author Contributions

AP was responsible for study design, contributed to treatment administration, data collection, analysis, and writing of the manuscript. EH and BC contributed to data collection and treatment administration. TC organized the dietary management. DA contributed to study design and data analysis. DC contributed to study design, treatment administration, and data collection. All authors were involved in writing the paper and approved the submitted and final versions.

## Conflict of Interest Statement

This research received no external funding. Dr. Poulton has shares in Glaxo-Smith-Klein. Dr. Champion has received honoraria for talks on diabetes from Novartis, MSD, and Astra Zeneca and travel support from Novartis to attend ESA in Boston in 2012. The other authors have no competing interests.
